# 463. Factors of Social Determinants of Health Associated with Length of Stay in COVID-19 Patients with Multimorbidity in Southwest Georgia, United States

**DOI:** 10.1093/ofid/ofab466.662

**Published:** 2021-12-04

**Authors:** Sahand Golpayegany, Sharmon P Osae, Geren Thomas, Henry N Young, Andrés F Henao Martínez, Carlos Franco-Paredes, Daniel B Chastain

**Affiliations:** 1 University of Georgia College of Pharmacy, Albany, Georgia; 2 John D. Archbold Memorial Hospital, Thomasville, Georgia; 3 University of Colorado Anschutz Medical Campus, Aurora, Colorado; 4 University of Colorado Denver, School of Medicine, Aurora, CO

## Abstract

**Background:**

Previous studies have observed that multimorbidity, defined as two or more comorbidities, is associated with longer lengths of stay (LOS) and higher mortality in patients with COVID-19. In addition, inequality in social determinants of health (SDOH), dictated by economic stability, education access and quality, healthcare access and quality, neighborhoods and built environment, and social and community context have only added to disparities in morbidity and mortality associated with COVID-19. However, the relationship between SDOH and LOS in COVID-19 patients with multimorbidity is poorly characterized. Analyzing the effect SDOH have on LOS can help identify patients at high risk for prolonged hospitalization and allow prioritization of treatment and supportive measures to promote safe and expeditious discharge.

**Methods:**

This study was a multicenter, retrospective analysis of adult patients with multimorbidity who were hospitalized with COVID-19. The primary outcome was to determine the LOS in these patients. The secondary outcome was to evaluate the role that SDOH play in LOS. Poisson regression analyses were performed to examine associations between individual SDOH and LOS.

**Results:**

A total of 370 patients were included with a median age of 65 years (IQR 55-74), of which 57% were female and 77% were African American. Median Charlson Comorbidity Index was 4 (IQR 2-6) with hypertension (77%) and diabetes (51%) being the most common, while in-hospital mortality was 23%. Overall, median length of stay was 7 days (IQR 4-13). White race (-0.16, 95% CI -0.27 to -0.05, p=0.003) and residence in a single-family home (-0.28, 95% CI -0.38 to -0.17, p< 0.001) or nursing home/long term care facility (-0.36, 95% CI -0.51 to -0.21, p< 0.001) were associated with decreased LOS, while Medicare (0.24, 95% CI 0.10 to 0.38, p=0.001) and part-time (0.35, 95% CI 0.13 to 0.57, p=0.002) or full-time (0.25, 95% CI 0.12 to 0.38, p< 0.001) employment were associated with increased LOS.

**Conclusion:**

Based on our results, differences in SDOH, including economic stability, neighborhood and built environment, social and community context, as well as healthcare access and quality, have observable effects on COVID-19 patient LOS in the hospital.

**Disclosures:**

**All Authors**: No reported disclosures

